# Recurrent metabolic alkalosis following ketone body treatment of adult mitochondrial trifunctional protein deficiency: A case report

**DOI:** 10.1002/jmd2.12309

**Published:** 2022-06-25

**Authors:** Nina N. Stolwijk, Mirjam Langeveld, Bart A. W. Jacobs, Liffert Vogt, Jorien A. Haverkamp, Sacha Ferdinandusse, Carla E. M. Hollak

**Affiliations:** ^1^ Medicine for Society Amsterdam UMC location University of Amsterdam Amsterdam The Netherlands; ^2^ Department of Endocrinology and Metabolism Amsterdam UMC location University of Amsterdam Amsterdam The Netherlands; ^3^ Department of Pharmacy and Clinical Pharmacology Amsterdam UMC location University of Amsterdam Amsterdam The Netherlands; ^4^ Division of Nephrology, Department of Internal Medicine Amsterdam UMC location University of Amsterdam Amsterdam The Netherlands; ^5^ Laboratory Genetic Metabolic Diseases, Department of Clinical Chemistry Amsterdam UMC location University of Amsterdam Amsterdam The Netherlands

## Abstract

Recent studies have reported the potential for the therapeutic use of ketones in the form of ketone salts (KSs) in pediatric patients with fatty acid oxidation disorders (FAODs). We report a case of ketone salt administration in an adult patient with mitochondrial trifunctional protein deficiency (MTPD), an ultra‐rare inborn error of the fatty acid metabolism. This patient was treated with oral KSs during an episode of sepsis of unknown origin. Before KS supplementation was initiated, he had developed severe rhabdomyolysis as well as a respiratory insufficiency that did not respond to emergency treatment aimed at stabilizing the metabolic decompensation by promoting anabolism. Therefore, KS supplementation was attempted twice to support his energy production and help regain metabolic stability. In both instances, KS supplementation led to a considerable metabolic alkalosis, which prompted its discontinuation. This adverse event could have been caused by an increase in extracellular sodium load due to KS administration. Therefore, the clinical applicability of KSs in adults may be limited. Alternative chemical forms of beta‐hydroxybutyrate (βHB), such as ketone esters, might provide a more acceptable safety profile for future research into the therapeutic benefits of ketone body supplementation in adult patients with FAODs.

## 1 INTRODUCTION

Fatty acids are the preferred source of energy of the human heart. Furthermore, a significant proportion of energy in skeletal muscle is derived from fatty acids, which increases during fasting, exercise, and other causes of increased metabolic demand.^1^, ^2^ Fatty acid metabolism is impaired in patients with the ultra‐rare inborn error mitochondrial trifunctional protein deficiency (MTPD, OMIM #609015). Patients with MTPD can develop cardiomyopathy, recurrent hypoketotic hypoglycemia and metabolic acidosis as well as episodic rhabdomyolysis, peripheral neuropathy, and retinopathy.^3^, ^4^ MTPD is caused by impaired functioning of the multi‐enzyme complex mitochondrial trifunctional protein (MTP). As its name suggests, MTP has three enzymatic subunits: long‐chain enoyl‐CoA hydratase, long‐chain 3‐hydroxyacyl‐CoA dehydrogenase (LCHAD), and long‐chain thiolase (LCTH).^5^, ^6^ These three enzymes catalyze the final steps of mitochondrial long‐chain fatty acid beta oxidation (FAO).

There is no curative treatment for MTPD and management is aimed at preventing metabolic crises and complications and optimizing exercise tolerance. Most MTPD patients are prescribed a diet that is rich in carbohydrates and protein and low in long chain fatty acids (10–15% of total energy intake for patients with severe disease).^7^, ^8^ The diet can be supplemented with even chain medium‐chain triglycerides (MCT), since metabolism of medium chain fatty acids is not impaired. More recently, odd chain fatty acid such as triheptanoin has been used in the treatment of long chain fatty acid oxidation disorders.^9^ Despite these management strategies, patients still suffer from significant disease symptoms, including reduced exercise capacity and metabolic crises.^10^, ^11^

Administration of exogenous ketone bodies has also been suggested as a treatment strategy for fatty acid oxidation disorders (FAODs).^12^, ^13^ In healthy individuals, the hepatic mitochondrial beta‐oxidation incites ketogenesis in the fasted state, producing ketone bodies as an alternative energy source. In patients with FAODs, including MTPD, impaired mitochondrial beta‐oxidation limits this capacity.^14^ Exogenous ketones could therefore provide therapeutic benefits as an alternative energy source, independent of beta‐oxidation of fatty acids. This hypothesis is supported by the improved performance observed in healthy athletes following the ingestion of ketones, which substantiates their proposed functioning as an alternative energy source.^15^, ^16^

The therapeutic use of ketones in the form of ketone salts (KSs) in metabolic disorders has been previously described, most extensively in children suffering from multiple acyl‐CoA dehydrogenase deficiency (MADD, OMIM #231680).^12^, ^17^, ^18^ Ketone salts are comprised of beta‐hydroxybutyrate (βHB) and a mineral (calcium, sodium, potassium, magnesium, or a mixture of these) and are only available as food supplements. We describe the clinical course of a MTPD patient who was unsuccessfully treated with oral KSs.

## 2 MATERIALS AND METHODS

This case presentation is based on a retrospective data analysis of a single patient with MTPD. Enzymatic studies (LCHAD and LCTH activity measurements in lymphocytes and fibroblasts, and long chain‐FAO flux analysis in fibroblasts) were performed as previously described.^19^ Genetic analysis was done by performing Sanger sequencing of all exons and flanking intronic sequences of the *HADHA* and *HADHB* genes.

### 2.1 Products

Two KS products were administered. First, a pharmacy‐compounded liquid formulation of racemic D,L‐βHB sodium salt was used, with a ratio D‐βHB:L‐ βHB of 1:1. The D‐ and L‐βHB isoforms' pharmacodynamical properties differ, with higher D‐βHB uptake in blood and tissues and L‐ βHB appearing more capable of passing the blood brain barrier.^20^ Since pharmaceutical grade βHB is unavailable, analytical grade sodium βHB was used as the active pharmaceutical ingredient for the oral solution (provided by University Medical Centre of Groningen [UMCG]). The identity of the analytical grade βHB powder was confirmed by the UMCG pharmacy department using infrared spectrophotometry and melting point analysis. This sodium‐D,L‐βHB oral solution was prepared at a final concentration of 593.3 mg/ml in water. The second product was a commercial food supplement containing a 1:1 racemic mixture of D,L‐ βHB sodium and potassium salt (KetoForce by KetoSports) with a βHB concentration of 390 mg/ml.

## 3 CASE PRESENTATION

### 3.1 Patient history and clinical presentation

A 24‐year old male patient was diagnosed with MTPD at the age of 5 years. After uneventful pregnancy and birth, progressive muscle weakness and a sensorimotor axonal neuropathy developed from the age of 2 years onwards. His condition worsened in the following years, with decreasing muscle function and episodes of vomiting and rhabdomyolysis. Investigations at the age of four showed an acylcarnitine profile compatible with MTPD. The diagnosis was confirmed by enzymatic analysis of the LCHAD and LCTH activities both in lymphocytes and fibroblasts, which were markedly reduced compared to the reference values. Long chain‐FAO flux in fibroblasts was reduced to 43% of controls. At 6 years of age, molecular testing revealed compound heterozygous *HADHA* variants (c.556C>G, p.Gln186Glu and c.1392 + 1G>A*)*. The previously reported c.1392 + 1G>A variant results in a splicing defect^21^_._ The missense variant c.556C>G has not been reported in other patients, is not present in the gnomAD database, and was classified as variant of unknown significance. Intensive dietary management was started, facilitated by the placement of a PEG‐tube. In recent years, these dietary measures consisted of daily overnight feeding (500 kcal of which 13 g/day MCT) and an adherence to a mild total fat restriction (approx. 25% of total daily energy intake), resulting in stable disease. Despite wheelchair dependency, related to his muscle weakness and polyneuropathy, he had a normal social and work life.

At 24 years old, this patient was admitted to hospital following an episode of febrile illness with shortness of breath, fatigue, and muscle pain. Emergency treatment was initiated immediately, consisting of IV infusions of glucose (10%, 2–4 L) and saline (NaCl 0.9%, 2 L, electrolyte‐based titration), additional enteral nutrition (day of admission: home/hospital nutrition combined; admission day 2–15: total caloric intake 2400–3706 kcal/day (1–1.5 times total energy expenditure [TEE, calculated using WHO equation, includes IV glucose], composed of 66–77% carbohydrates, 8.0–16% protein and 11–16% total fat of which MCT 40 g/day and LCT 6.8 g/day) and insulin (insulin pump from day 1–8, target glucose 6–9 mmol/L). Arterial blood gas analyses revealed respiratory acidosis as a consequence of high CO_2_, related to a severe respiratory muscle weakness, assessed by neurological exam. Hence, following admission to the ICU, respiratory failure required initiation of mechanical ventilation. Repeated analysis for SARS‐Cov2 was negative and no other infectious agents were identified, even though CRP was significantly elevated at time of admission (141 mg/L, ref 0–5) and he was treated with broad spectrum antibiotics (cefotaxime and gentamicin, the latter substituted by ciprofloxacin on day 2).

**FIGURE 1:**
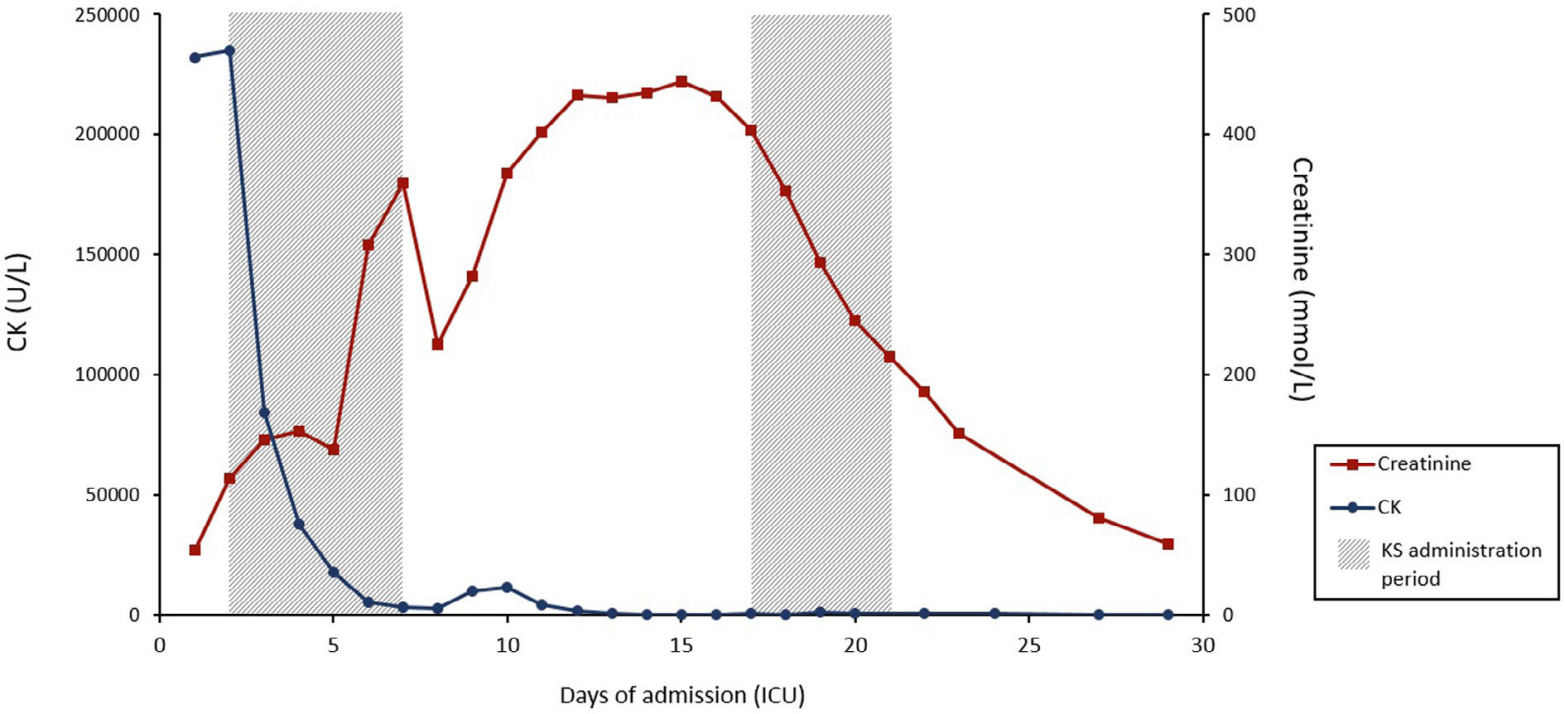
CK and creatinine levels during ICU admission

During admission, rhabdomyolysis initially worsened, as can be seen in acute decompensation despite adequate measures, with CK levels rising above 200 000 U/L (see Figure 1). This resulted in acute kidney injury (AKI), requiring continuous veno‐venous hemofiltration (CVVH) for the first 9 days of admission (day 1 bicarbonate, day 2–9 citrate CVVH). Although an echocardiogram did reveal regional wall motion abnormalities, left ventricular function was not diminished. Attempts to extubate the patient failed due to severe muscle weakness. For several days, his condition remained critical while rhabdomyolysis persisted. Therefore, the use of KSs was initiated to support his energy production and help regain metabolic stability, reduce muscle breakdown and improve (respiratory) muscle strength.

### 3.2 Treatment outcomes

**FIGURE 2:**
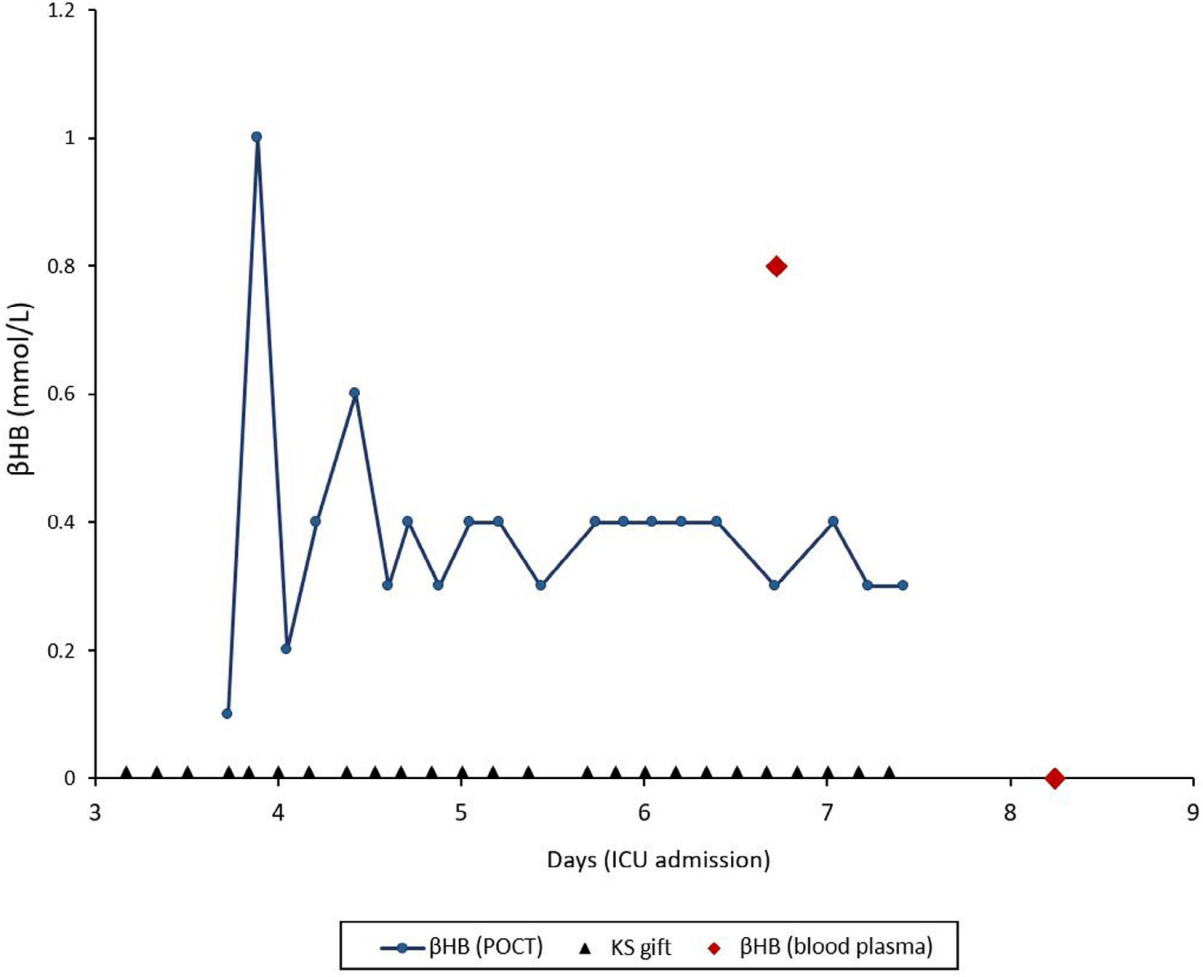
βHB levels 1 h after ketone salt administration (day 2–7)

**FIGURE 3:**
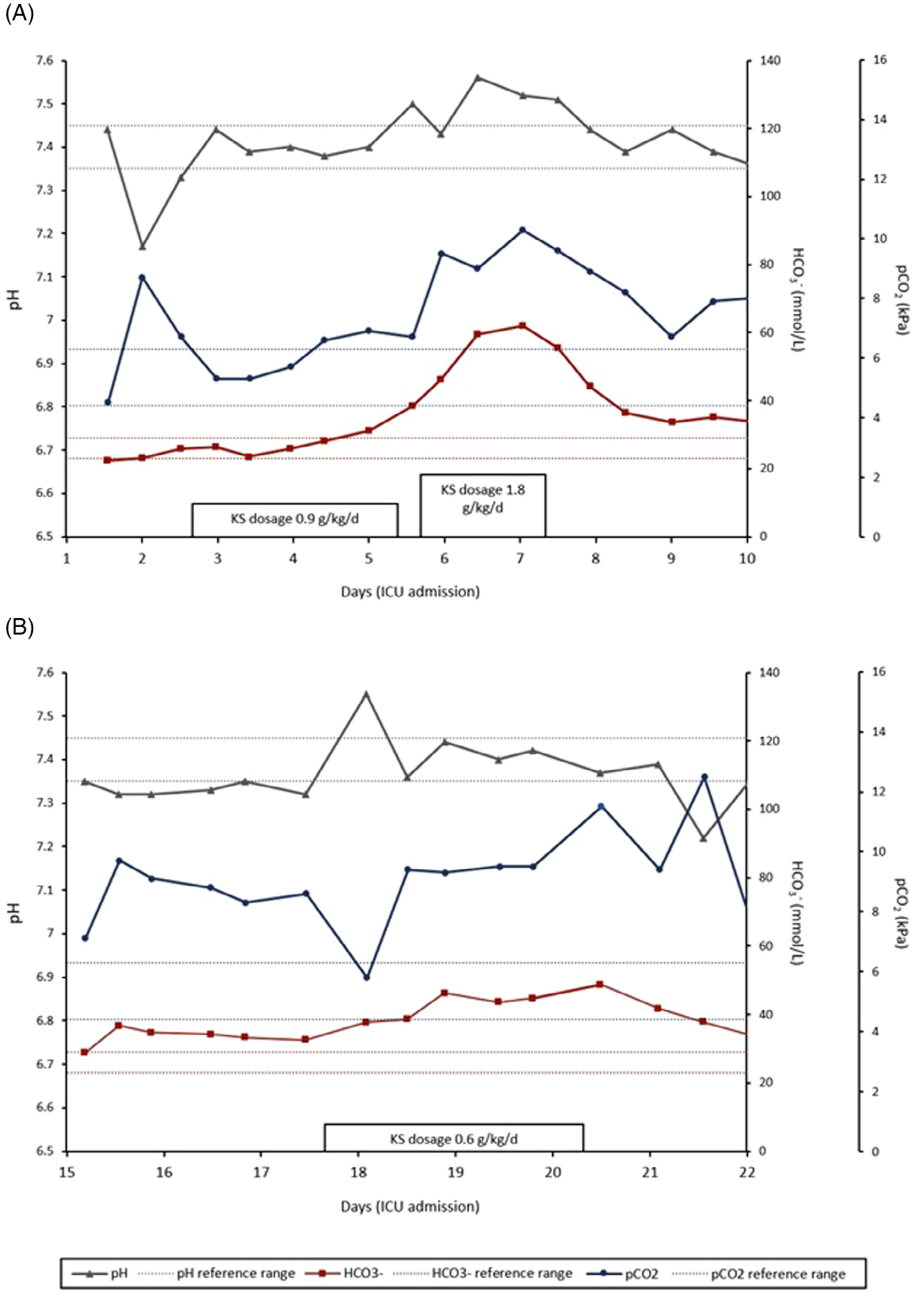
Blood pH, bicarbonate‐, and pCO_2_ levels following daily administration of A., sodium βHB as a hospital pharmacy preparation (day 2–17) and B., sodium and potassium βHB (day 17–20)

Two days after admission to the ICU, the administration of exogenous ketones in the form of KSs was first initiated (sodium βHB as a hospital pharmacy preparation, initial dosage 0.9 g/kg/day, an absolute dosage of 59 g/day βHB and 12.9 g/day sodium, administered though PEG tube in six doses). The βHB concentration was recorded during this first treatment period with capillary test strips 1 h before and after each administration (see Figure 2, βHB ref 0.0–0.2 mmol/L, measured with StatStrip Xpress by A.Menarini), as well as twice by blood chemistry following venipuncture. On the third day of treatment, the KS dosage was increased to 1.8 g/kg/day (absolute dosage of 117 g/day βHB and 25.6 g/day sodium). By the fifth day of treatment, a severe alkalosis became apparent, eventually necessitating cessation of KS therapy (see Figure 3A). Since bicarbonate as well as pCO_2_ levels rose during treatment, while the anion gap remained steadily low (AG before start of treatment 4.2; day after cessation 4.9, ref 8–11 mmol/L), this was deemed to be a metabolic alkalosis caused by exogenous alkali. Simultaneously, a hypernatremia and hypochloremia were observed (peak Na^+^ 155, ref 135–145 mmol/L; concurrent Cl^−^ 82, ref 98–107 mmol/L). Shortly after discontinuation of KSs, the patient's blood pH and bicarbonate levels normalized.

Ten days later, the patient's condition had not improved. Therefore, a reduced dosage of KS was attempted with a KS food supplement (sodium and potassium βHB, 0.6 g/kg/day, absolute dosages of 41 g/day βHB and 6.6 g/day sodium and potassium respectively, in six doses). However, again, recurrence of metabolic alkalosis led us to discontinue the treatment before potential clinical improvements could be observed (see Figure 3B). With continuation of diet and supportive care, the patient could eventually be weaned successfully after a month. At this time, he is gradually recovering with intensive rehabilitation.

## 4 DISCUSSION

We report the case of a 24‐year‐old patient with MTPD who was treated with ketones in the form of KSs during an episode of severe metabolic decompensation with rhabdomyolysis and muscle weakness triggered by an infection. In both instances, KSs induced metabolic alkalosis and discontinuation was required. After the withdrawal of KSs, acid–base derangements quickly improved. Although citrate and/or bicarbonate CVVH might induce a similar effect, CVVH was stopped before the second episode of KS therapy and thus does not explain the observed metabolic alkalosis. Additionally, recurrent metabolic alkalosis following KS therapy has been previously described in a boy with MADD^22^; Dosages were within the range administered our patient (0.6–1.8 g/kg/day). In healthy volunteers, an increase in blood pH was also reported following KS consumption (although within the normal range).^23^

The mechanism though which KSs might induce alkalosis is not yet fully understood. One potential explanation is the alkalizing effect of the salt load.^22^ Stewart's acid–base approach has been proposed to understand this mechanism.^24^, ^25^ In this approach, the strong ion difference (SID) depicts the net balance between strong, fully dissociated cations, and strong anions. Increases in the strong ion differences, like those induced by an high Na^+^ load following KS ingestion, increase the SID, which in turn raises the pH.^24^ This hypothesis is substantiated by the hypernatremia and concurrent hypochloremia observed during the first period of high‐dosage KS therapy, while the anion gap remained unchanged.

The alkalizing effect of KSs has also been attributed to βHB itself, which was thought to fully dissociate and act as a conjugate base.^23^ The opposing effect of ketone esters (KEs) which also provide βHB, however, contradicts this. KEs induced mild acidosis shortly after administration and did not significantly affect blood pH in the long term.^23^, ^26^ Attributing the alkalizing effect of KSs to its cation load is further substantiated by KS toxicology studies in mice, which found a quick intracellular uptake of βHB from the circulation, whereas the accompanying Na^+^ remained.^27^ This cation load may limit the tolerability of KSs, since dosages needed to treat adults with FAODs are relatively high. Although previous studies have shown therapeutic benefits of KS supplementation in pediatric patients, alternative forms of βHB without a salt load (e.g., KEs) might be more suitable to adults.^28^

Another concern illustrated by this case is the lack of (access to) high quality therapy products for MTPD. Triheptanoin, a synthetic odd‐carbon medium‐chain triglyceride that can be used as energy substrate replacement therapy for patients with long‐chain FAODs, could also have been tried in this setting.^9^ However, as is the case for βHB, no pharmaceutical grade product is available in Europe. To advance treatment outcomes, improving this status quo is paramount. At present, exogenous βHB administration could only be achieved by administration of analytical grade compounds or food supplements. This clinical reality highlights the urgent need for the development of high quality alternative forms of βHB—potentially without high salt loads—in order to study the clinical efficacy and safety profile of βHB in adult patients with FAODs.

## AUTHOR CONTRIBUTIONS

N.N.S., M.L, and C.E.M.H. conceptualized the case report. N.N.S. collected the data, wrote the draft manuscript, and created the Figures. M.L., B.A.W.J, and L.V. analyzed the data, critically reviewed the manuscript, and edited where needed. J.A.H. collected and analyzed the dietary data and critically reviewed the manuscript. S.F. analyzed the diagnostic data and critically reviewed the manuscript. C.E.M.H. oversaw the general direction of the article and critically reviewed the manuscript.

## CONFLICT OF INTEREST

Outside of submitted work, M.L. reports to be involved in pre‐marketing studies with Genzyme, Protalix, and Idorsia. Financial arrangements are made through AMC Research BV. C.E.M.H. reports to be involved in pre‐marketing studies with Genzyme, Protalix, and Idorsia. Financial arrangements are made through AMC Research BV. N.N.S., B.A.W.J., L.V., J.A.H., and S.F. have no conflicts of interest to disclose.

## ETHICS APPROVAL

As this is a single descriptive case report, ethics board review was not required by our institution.

## PATIENT CONSENT STATEMENT

The patient has provided written informed consent for the publication of this case report.

## Data Availability

The data that support the findings of this study are available from the corresponding author, C.E.M.H, upon reasonable request.
